# Where art meets neuroscience: a new horizon of art therapy

**DOI:** 10.3325/cmj.2014.55.73

**Published:** 2014-02

**Authors:** Lukasz M. Konopka

**Affiliations:** Department of Psychiatry, Loyola Medical Center, Maywood, IL, USA and Yellowbrick Consultation and Treatment Center, Evanston, IL, USA

Advances in human brain imaging help us evaluate brain functions from many perspectives. We can define structural brain differences between individuals with various disorders, such as adult schizophrenia (1) and childhood, autistic-spectrum disorders (2). From these studies, we can posit hypotheses regarding structure, and how structure relates to symptoms. However, often we find it difficult to assign causality to such findings. For example, we can ask whether structural anomalies cause the symptoms or whether symptoms drive the abnormal brain structures. Nevertheless, we can confidently say that the brain’s structure changes as a consequence of illness and activity, eg, imaging data show that with proper rehabilitation, the injured brain rewires and recovers its function (3). We label this process “brain plasticity.” Various fields use the concept of brain plasticity. One such very exciting, emerging field involves the study of art and the brain, or art therapy (4). Originally, art therapy used pure art concepts, void of scientific inquiry. Now, slowly, it is embracing scientific thinking by using abundant neuroscientific data and the objective tools of scientific investigation. For years, we recognized that art-making allowed one to reframe experiences, reorganize thoughts, and gain personal insights that often enhanced one’s quality of life. Art therapy has gained popularity because it combines free artistic expression with the potential for significant therapeutic intervention. Although based on subjective data and testimonies, various artistic disciplines have helped patients with diverse disorders that include developmental or acquired, medical, and/or psychiatric conditions (5,6).

To utilize nonstandard, medical therapies within the well-established medical model, we must demonstrate the utility and efficacy of novel tools and approaches. The scientific method is one way we can demonstrate that art and art therapy modify the brain’s physiology and structure and lead to a more flexible, adaptable individual. Moreover, if we want to validate non-standard approaches, such as art therapy, we need more studies to assess their effects on brain function. As we might imagine, it is very difficult to define art and its optimal therapeutic uses. Naturally, as a new field, art therapy is trying to define its territory and claim its domain within brain science. To gain acceptance and credibility from the medical establishment, art therapy is, seemingly, hoping to assign unique artistic processes to specific brain structures, but the specific brain effects of the artistic process are difficult to study. Nevertheless, through neuroscience, art therapy is attempting to locate particular brain areas or activity patterns that may be devoted exclusively to art-making (7,8). Yet, this specificity presents a problem – the brain does not distinguish between the processes used to create a scientific invention and a work of art – the brain undergoes identical activity sequences and manipulations (9,10).

At the outset, an artist may wish to express an idea and a scientist may hope to develop a new treatment or novel molecule. Next, both artist and scientist choose their tools. Then, both experiment, and, eventually, create a final product. At the system level, the brain is unaware of the anticipated outcome, ie, a new pharmaceutical agent or a sculpture. If we accept that scientific and artistic processes use congruent networks, we can assume that artists and scientists use very similar brain processes to deploy their conceptualizations (11). As such, in terms of therapy, there is no difference between using scientifically validated novel art therapy and other current standard therapeutic interventions. Treating human pathology using art gives us a tremendous alternative unique and novel option for engaging brain networks that enhance the way the brain processes information, incorporates external and internal data, and develops new efficient brain connections. Ultimately, our goal is for humans to become better adapted to their defined environments. It is quite evident that scientists, clinicians, and artists must come together to share and discuss their experiences. Their interaction can lead to novel communication and cooperation. Clearly, at the brain level, any intervention’s goal is the dynamic enhancement of emotion, cognition, and executive flexibility so that one fully participates in life and avails oneself of the experiential and hereditary gifts in his or her environment (12). Ultimately, we hope to integrate all disciplines without prejudice and develop novel therapies that optimize the treatment of mental illness.
